# Peer assessment of professionalism in undergraduate medical education

**DOI:** 10.1186/s12909-020-02412-x

**Published:** 2020-12-11

**Authors:** Vernon R. Curran, Nicholas A. Fairbridge, Diana Deacon

**Affiliations:** Office of Professional and Educational Development, Faculty of Medicine, Memorial University, St. John’s, Newfoundland A1B 3V6 Canada

**Keywords:** Peer assessment, Undergraduate medical education, Professionalism

## Abstract

**Background:**

Fostering professional behaviour has become increasingly important in medical education and non-traditional approaches to assessment of professionalism may offer a more holistic representation of students’ professional behaviour development. Emerging evidence suggests peer assessment may offer potential as an alternative method of professionalism assessment. We introduced peer assessment of professionalism in pre-clerkship phases of undergraduate medical education curriculum at our institution and evaluated suitability of adopting a professional behaviour scale for longitudinal tracking of student development, and student comfort and acceptance of peer assessment.

**Methods:**

Peer assessment was introduced using a validated professional behaviours scale. Students conducted repeated, longitudinal assessments of their peers from small-group, clinical skills learning activities. An electronic assessment system was used to collect peer assessments, collate and provide reports to students. Student opinions of peer assessment were initially surveyed before introducing the process, confirmatory analyses were conducted of the adopted scale, and students were surveyed to explore satisfaction with the peer assessment process.

**Results:**

Students across all phases of the curriculum were initially supportive of anonymous peer assessment using small-group learning sessions. Peer scores showed improvement over time, however the magnitude of increase was limited by ceiling effects attributed to the adopted scale. Students agreed that the professional behaviours scale was easy to use and understand, however a majority disagreed that peer assessment improved their understanding of professionalism or was a useful learning experience.

**Conclusions:**

Peer assessment of professional behaviours does expose students to the process of assessing one’s peers, however the value of such processes at early stages of medical education may not be fully recognized nor appreciated by students. Electronic means for administering peer assessment is feasible for collecting and reporting peer feedback. Improvement in peer assessed scores was observed over time, however student opinions of the educational value were mixed and indeterminate.

## Background

The assessment of professionalism and students’ professional behaviour has become an increasingly important part of the medical school curriculum with peer assessment emerging as a possible, alternative method for providing formative peer feedback to students regarding their professional behaviour [1–4]. Peer assessment is defined as assessment by and of individuals who have attained the same general level of training or expertise, exercise no formal authority over each other, and share the same hierarchic status in an institution [5]. Peer assessment allows students to assume the assessor role, while also enabling students to gain insight into their own performance and improve the quality of their self-assessment [6]. Peer assessments can be used for summative or formative evaluation purposes, although more commonly peer assessment of professional behaviours has been used for formative feedback purposes in medical education [2, 6–8].

Emerging evidence suggests that peer assessment may be effective in promoting professionalism and can provide valuable formative feedback on professional behaviors and skills [2, 9, 10]. Studies have demonstrated it can be a reliable method for assessing the humanistic/psychosocial dimensions of clinical performance [1, 2, 8]. Apart from supporting student learning, assessment and feedback on professional behaviours may also offer opportunities for early detection, monitoring and/or timely remediation of students who display difficult or challenging behaviours [10, 11]. As peers are able to observe one another regularly over a wide range of circumstances, peer assessment may also provide information regarding student performance that is not measured by other traditional evaluation methods [1, 12–15].

Donald Schon’s theory of the ‘reflective practitioner’ advocates for a form of reflective practice by which practitioners are thoughtful towards reflection on their practices and the learning that may arise from that reflection on their actions [16, 17]. According to Hulsman et al. [8] peer-assessment also reflects an ‘assessment-for-learning paradigm’ that is rooted within principles of social constructivist theories of learning. In many ways, the reported benefits of peer assessment in medical education mirror many of the key characteristics of Schon’s notion of the ‘reflective practitioner’, including that it may confer an improved ability to engage in self-reflective practice, develop greater self-awareness and increased critical reasoning skills [2, 7, 8, 10, 14, 18].

Sadler and Good [19] suggest that peer assessment also fosters ‘metacognition’, which is knowledge or awareness of one’s own learning processes, and as learners become more aware of performance expectations they internalize this understanding and apply it to their future work to improve their own performance. Engaging in peer feedback may also increase student motivation by fostering a greater sense of accountability and ownership of learning [10]. Generally, students find peer assessment acceptable and growing evidence suggests peer assessment can predict future academic performance, provide medical students with reliable feedback about professionalism and may enhance future professional behaviour [2, 3, 15, 18].

Students prefer peer assessments that are conducted in a supportive environment, are anonymous, provide immediate feedback, focus on both unprofessional and professional behaviors, and use the assessments formatively. However, despite the benefits of peer assessments, few successful peer assessment systems have been implemented and a reluctance of students to participate in peer assessments have been noted [3]. We sought to introduce and evaluate the feasibility, educational effect and acceptability of the use of peer assessment of professional behaviours during the pre-clerkship phase of an undergraduate medical education curriculum. Our specific evaluation questions included:

- Does a professional behaviours scale previously validated for cross-sectional use retain validity and reliability for longitudinal peer assessment of professionalism amongst undergraduate medical students?
- What are the educational benefits of peer assessment of professionalism in pre-clerkship phases of undergraduate medical education curriculum?
- What is the perceived educational value of peer assessment of professionalism amongst undergraduate medical students?

## Methods

Memorial University of Newfoundland’s four-year Doctor of Medicine (MD) degree is organized into four phases with first year consisting of phases 1 and 2, second year phase 3, and the final 2 years phase 4 (clerkship). Phase one covers normal health and development, phase two covers acute reversible or modifiable health issues and phase three covers chronic disease. In each of phases 1–3, students complete a clinical skills course during which they learn in small groups. Memorial University introduced a peer assessment process in the pre-clerkship phase (phases 1–3) of our undergraduate medical education curriculum that aligned with the small-group, clinical skills learning in our Clinical Skills I – III courses across phases 1–3 of the curriculum. We conducted a mixed cross-sectional and time-series evaluation study design. Initially, we started with a cross-sectional survey in early 2017 of all undergraduate medical students across all phases to identify perceived barriers to peer assessment program acceptability and implementation. The survey findings were used to inform the design of the peer assessment system and its introduction in the undergraduate curriculum. Next, we introduced a longitudinal peer assessment program beginning in 2017, repeating with classes entering in 2018 and 2019. Our evaluation involved an aggregate summary and analysis of peer assessment scores across these three class cohorts at 4 time points (TP) reflecting the 4 semesters of clinical skills held through phases 1 to 3. Finally, the evaluation also included an anonymous survey of students that was distributed at the midpoint and end of the program.

The pre-implementation survey used a six item, checklist-based questionnaire that asked students for their opinion on: degree of anonymity of feedback, appropriate content domains for peer assessment, peer assessors, access to peer feedback, and whether peer assessment should be formative or summative. The survey results were used to inform the peer assessment system design. Peer assessment was introduced using the Dannefer et al. [1] ‘peer assessment of professional behaviours’ scale that includes 15 Likert-style items rated from 1 = “unsatisfactory” to 5 = “exceptional” which cluster into two subscales; ‘Professional Work Habits’ and ‘Interpersonal Habits’. Students were also asked to comment on the strengths and weaknesses regarding professional attributes for peers assigned to their specific clinical skills small learning groups. This scale was administered to the students electronically using the One45 software system that permitted peer assessment reports to be provided to each student with mean scores and range from assessors, and written feedback on strengths and weaknesses.

Participation and completion of the peer assessment was required, and students were oriented to the process of peer assessment and the use of the peer assessment scale at the beginning of each course, and provided with guidance on the nature of feedback that would be most constructive for their peers. Final peer assessment submissions were screened for derogatory and/or inflammatory comments, and students were offered the opportunity to meet with a faculty member to review and/or discuss peer assessment feedback for further coaching support and assistance. Due to practical constraints imposed by the existing delivery of the clinical skills course curricula, the effective group sizes of small groups in the clinical skills courses decreases across phases 1–3, and group membership also changes, but does remain constant for each clinical skills course I – III. Our evaluation encompassed an analysis of the longitudinal, repeated scores from the peer assessments using the Dannefer et al. [1] scale. Exploratory factor analyses was conducted to confirm construct validity of the original scale as adopted for medical student peer assessment. Cronbach’s α was examined to assess internal consistency and two-way random inter-class correlation was conducted to evaluate required number of peer assessors to ensure reliability in a peer assessment setting.

A web-based evaluation survey was also distributed to students at the end of each academic year to evaluate satisfaction with the adopted peer assessment process and methodology. The survey included ten Likert-type scale items rated from 1 = “strongly disagree” to 5 = “strongly agree”, with an additional N/A option. Students were asked to rate their opinion on: adequate preparation for peer assessment, ease of completion and understanding of the scale, confidence in ability to rate peers, effect of peer feedback on understanding of professionalism, fairness and overall usefulness of peer assessment as a learning experience. They were also invited to provide open-ended comments on positive aspects of peer assessment and ways it could be improved and responses were summarized into broad themes using thematic analysis.

## Results

The pre-implementation student survey returned an overall response rate of 47.8% (*n* = 153) from all undergraduate classes enrolled as of 2017. The vast majority (80.4%, *n* = 123) of students supported a totally anonymous peer feedback process, but raised concerns with small group sizes constraining or preventing assured anonymity. Student respondents supported only the assessed individual having access to the feedback (89.5%, *n* = 137) and most (81.0%, *n* = 124) supported its use as a formative assessment process. Students were also supportive of their peers assessing professionalism (86.3%, *n* = 132), leadership (71.2%, *n* = 109) and performance in small group activities (70.6%, *n* = 108). Majority of students were supportive of classmates from small group learning sessions providing peer assessment (66%, *n* = 101).

Table 1 summarizes aggregated peer-assessor scores across the Dannefer et al. [1] professional behaviours scale. The 241 participating, pre-clerkship medical students submitted 2756 peer assessments with the largest missing data (non-response) (*n* = 113, 4.1%) for the item asking whether they “would refer this future physician to a family member”, representing 19.2% of all missing data. As with the initial analysis conducted by Dannefer et al. [1], the item on referrals was dropped based on the high degree of missing data and negative student feedback. Perfect scores of “exceptional” across all 15 items were recorded in 1180 assessments, representing 44.6% of all peer submissions.

**Table 1:** Professional competency item scores

	Mean	Median	SD
1 Consistently well-prepared	4.73	4.75	0.32
2 Identifies and solves problems	4.75	4.75	0.28
3 Clearly explains reasoning processes	4.75	4.75	0.26
4 Demonstrates respect	4.87	5.00	0.24
5 Seeks to understand others	4.81	5.00	0.28
6 Takes initiative	4.63	4.67	0.37
7 Shares information with others	4.78	4.87	0.29
8 Seeks responsibility	4.80	5.00	0.27
9 Asks for feedback	4.73	4.75	0.30
10 Trustworthy	4.87	5.00	0.25
11 Admits mistakes	4.88	5.00	0.21
12 Dresses appropriately	4.93	5.00	0.16
13 Behaves appropriately	4.90	5.00	0.22
14 Thinks and works independently	4.83	5.00	0.23
15 Would refer family member to peer	4.83	5.00	0.27

*n* = 642 assessments, 241 students

**Fig. 1:**
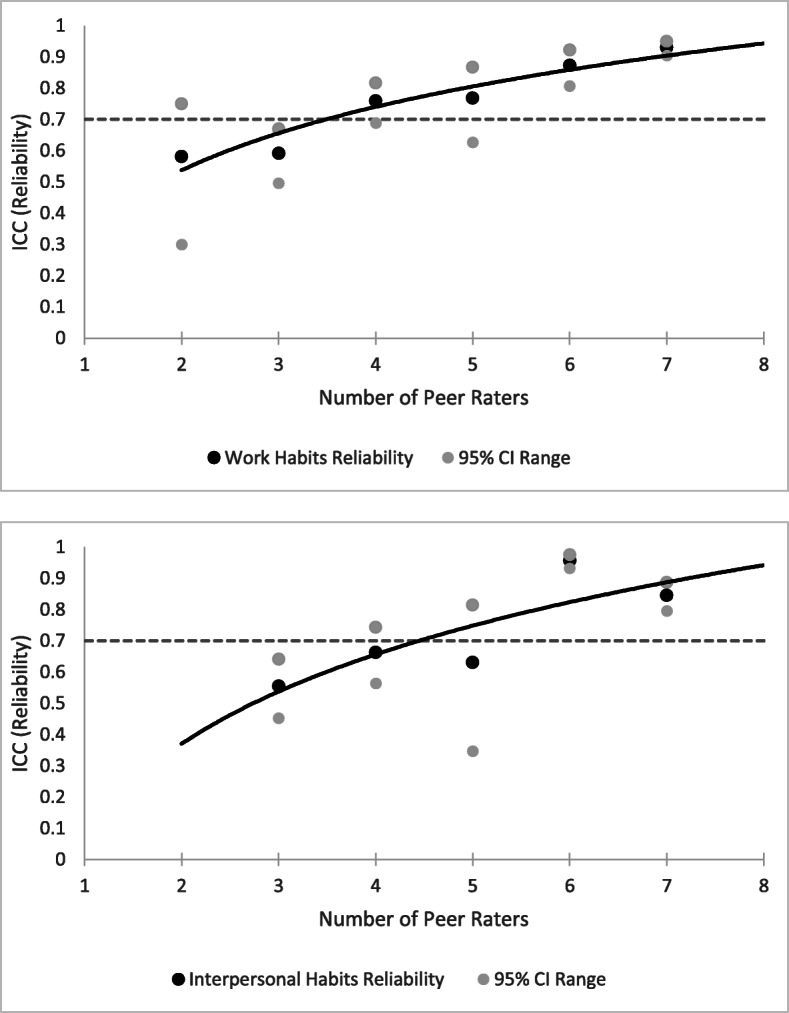
Inter-class correlation reliability analyses by group size. Data was binned by the number of assessors and 2-way random inter-class correlations (ICC) calculated per group size for each of the professionalism scales. The number of assessment groups per size category varied and arose from constraints during implementation, including variation in initial working group size and missed assessment submissions. The 95% confidence intervals of each ICC measure were plotted in gray and a reliability co-efficient of 0.7 was considered the threshold for consistent assessments

Table 2 summarizes the results of an exploratory factor analysis of the aggregated peer assessor scores. This analysis supported the original 2 dimensions of ‘Work Habits’ and ‘Interpersonal Habits’ of the Dannefer et al. [1] scale, which showed moderate to strong correlation (*r* = 0.63) between scales. Figure 1 summarizes reliability coefficients resulting from 2-way random Inter-Class Correlation analyses for consistency striated by group size. Reliability measures were plotted against the number of raters for both the ‘Work Habits’ and ‘Interpersonal Habits’ factor-weighted sub-scales, confirming that 6 or more peer assessors were required to exceed reliability targets with confidence.

**Table 2:** Factor analysis of professional competence scale items

	Factor pattern (weights)		Factor structure (correlations)	
	Work habits	Interpersonal habits	Work habits	Interpersonal habits
Identifies and solves problems	0.90		0.88	0.57
Thinks and works independently	0.86		0.87	0.56
Clearly explains reasoning processes	0.83		0.83	0.52
Consistently well-prepared	0.85		0.8	0.4
Seeks responsibility	0.76		0.86	0.64
Takes initiative	0.81		0.8	0.49
Shares information with others	0.32	0.53	0.66	0.74
Asks for feedback	0.35	0.51	0.67	0.73
Seeks to understand others		0.92	0.53	0.89
Demonstrates respect		0.92	0.48	0.85
Admits mistakes		0.80	0.58	0.85
Trustworthy		0.87	0.54	0.86
Dresses appropriately		0.52	0.39	0.56
Behaves appropriately		0.88	0.45	0.82
Cronbach’s α for each factor	0.91	0.91		
Variance explained for each factor	11.60%	55.90%		
Correlation between factors	0.63

The weighted ‘Work Habits’ and ‘Interpersonal Habits’ subscale scores were also analyzed over time using non-parametric Friedman tests (Table 3). Changes in ‘Work Habits’ and ‘Interpersonal Habits’ scores indicated significant changes over time, and Post-hoc analysis with Wilcoxon signed-rank tests demonstrated a significant increase in ‘Work Habit’ scores between time-points 3 and 4 (Z = − 6.62, *p* < 0.001) with gradual progression between other points showing significant increases from points 1 to 3 (Z = − 3.43, *p* = 0.001), and 2 to 4 (Z = − 4.69, *p* < 0.001). Post-hoc analysis with Wilcoxon signed-rank tests demonstrated significant increases in ‘Interpersonal Habits’ scores between time-points 1 and 2 (Z = − 3.94, *p* < 0.001) and 3 to 4 (Z = − 4.56, *p* = 0.001). Generally, the subscales demonstrate a capacity to track improvements over time, but are limited by ceiling effects per item and thus per subscale, restricting the potential for further improvement to the upper limits of the scale. Sixty nine students (*n* = 69, response rate 28.6%) completed the web-based student evaluation survey (Table 4). Overall, the ease of process for conducting peer assessment was viewed favourably by participants. Students agreed that the electronic rating system was easy to use (91.3%, *n* = 63) and the professional competence scale was easy to understand (78.2%, *n* = 54). The majority also agreed they were adequately prepared (67.2%, *n* = 43), however 32.3% (*n* = 22) did indicate they were not confident in their ability to rate peers and 36.8% (*n* = 25) disagreed that their peers were honest and responsible in their assessments. Respondents disagreed (30.3%, *n* = 20) or were unsure (33.3%, *n* = 22) about the fairness of peer assessment, and many disagreed (50.0%, *n* = 36) or were unsure (22.1%, *n* = 15) on whether providing peer assessments improved their understanding of professionalism. A majority (68.8%, *n* = 44) did not support the usefulness of peer assessment as a learning experience.

**Table 3:** Friedman analysis of peer assessment score improvement over time

	TP^a^ 1	TP 2	TP 3	TP 4	Friedman
Work Habits Score					
Mean	26.25	26.5	26.93	27.45	X2 (3)=52.07
Mean Rank	1.95	2.22	2.50	3.33	*P* < 0.001
Interpersonal Habits Score					
Mean	28.19	28.56	28.83	29.05	X2 (3)=56.23
Mean Rank	1.73	2.38	2.75	3.15	*P* < 0.001

^a^Time Point (TP) 1–4 map to each semester of clinical skills held in phases 1–3 of the curriculum

**Table 4:** Overall student satisfaction with peer assessment

Item	Strongly disagree		Disagree		Neither agree nor disagree		Agree		Strongly agree	
	N	%	n	%	n	%	n	%	n	%
I was adequately prepared to participate in the peer assessment process.	2	3.1	3	4.7	16	25.0	35	54.7	8	12.5
It was easy to complete the peer assessments in One45.	0	0.0	1	1.4	5	7.2	43	62.3	20	29.0
The items on the professionalism scale were easy to understand.	2	2.9	8	11.6	5	7.2	43	62.3	11	15.9
I was confident in my ability to rate my peers using the scale provided.	6	8.8	16	23.5	17	25.0	22	32.4	7	10.3
I felt that peer raters were honest and responsible.	10	14.7	15	22.1	9	13.2	28	41.2	6	8.8
Providing peer assessment increased my understanding of professionalism.	18	26.5	16	23.5	15	22.1	16	23.5	3	4.4
Receiving peer assessment increased my understanding of my own professionalism.	16	23.9	15	22.4	14	20.9	17	25.4	5	7.5
The written comments were useful for my professional development.	16	23.5	13	19.1	14	20.6	19	27.9	6	8.8
The process of peer assessment was fair.	6	9.1	14	21.2	22	33.3	16	24.2	8	12.1
Overall, this was a useful learning experience.	15	23.4	12	18.8	17	26.6	15	23.4	5	7.8

Open-ended comments suggested students found the professionalism scale easy to complete and were encouraged by the peer assessment feedback provided. Students supported an educational focus on professionalism, expressed appreciation for the positive peer interactions and the reflective aspects of the assessment process introduced early into the program, but questioned the overall effectiveness or usefulness of the scale. Concerns were expressed regarding the functional lack of anonymity due to small group size. Students identified inappropriate scale items (such as “appropriate dress”), and a lack of constructive feedback as limitations. While they acknowledged and appreciated the positive feedback, student respondents suggested such encouragement was less useful to foster formative improvements and that negative feedback was avoided due to anonymity concerns within peer groups.

## Discussion

The successful introduction of peer assessment is dependent on a number of factors, including the type of method adopted, recipients of the peer assessment information and how it is used, and issues surrounding the anonymity and confidentiality of the feedback [5, 13]. Anonymity is an important consideration and frequently mentioned way to encourage genuine participation in peer assessment. Students believe that anonymity can protect both the student evaluator and the peer who is being evaluated, and reduce the prospect of disrupting teamwork and interpersonal relationships while promoting more candid and honest assessments [5, 9].

Medical students are generally positive about peer assessment of professional behaviours [9], although the literature is mixed regarding student acceptance and satisfaction with peer assessment [11]. Papinczak et al. [18] found students’ reported feeling uncomfortable carrying out peer assessment, while Dooley and Bamford [10] suggest that, for many students, the idea of directly evaluating one’s peers is at odds with traditional conceptualizations of the role of peers. It has been suggested that resistance to formative peer feedback is less common than summative peer assessment and student anxiety has been shown to decrease with time following early and repeated inclusion of peer assessment [10]. Medical students have reported that the sooner they are required to undertake peer assessment, the more accustomed they become to doing it [2, 10].

Studies also suggest that students’ opinions are mixed on whether peer assessment should be informal and optional or formal and required [5]. In the current study, we introduced peer assessment as formal, but formative assessment. However, student views appeared to change during peer assessment implementation with students reporting on evaluation surveys a preference for facilitated/guided face-to-face discussions of performance directly with peers. It may be that participation in the peer assessment process itself fostered confidence and willingness to directly engage peers in discussing professional competence.

A number of factors can influence the reliability of peer assessments, including: the number of relevant performances observed, the number of peers involved and the number of aspects of competence being evaluated [2]. Nofziger et al. [15] found peers can provide reliable, stable ratings of both work habits (e.g., preparation, problem solving initiative) and interpersonal attributes (e.g., truthfulness, respect, integrity, empathy), and ratings of work habits were also predictive of future measures of achievement such as clerkship grades and residency directors’ evaluations. The findings from our study reaffirm the Dannefer et al. [1] call for 6 or more raters for reliable use of the professional competency scales and confirm the scales’ validity and structure but do not support the professional behaviours scale for longitudinal tracking of development.

The modality for administering peers assessments can also vary and include electronic, paper-based, and/or face-to-face depending on assessor preference and convenience [2, 5]. It can be more convenient for assessors to log on to an online instrument, confidentiality is easier to maintain, and results can be collated and analysed quickly without the need for considerable administrative support [2, 9]. Our study findings suggest that students perceived an electronic delivery format as a feasible and acceptable means for administering, collating and reporting peer assessment feedback. Based on our evaluation findings, we would offer the following recommendations regarding adoption of a professional behaviour-type scale for peer-assessment of professionalism:

- Students must be oriented to the assessment scale to be used in peer assessment and understand the process by which peer assessment will be undertaken. Instruction must also be provided to students on how to provide constructive feedback to one’s peers.
- Peer assessment must be promoted to students as a positive learning and development experience, that mirrors the responsibility that will be expected of them during their professional medical practice.
- Small-group learning courses in which students are learning together in stable groupings for an extended period of time would be preferred context for applying peer assessment activities.
- Students should be asked to engage in a ‘reflective’, summative assessment activity following the peer assessment process to enable reflection on the experience, learnings and implications for future professional practice.

## Conclusions

The study findings suggest there was mixed acceptance of peer assessment of professional competence in pre-clerkship medical education. Students reported differing views of the value and usefulness of the process, whether that be as a peer assessor and/or receiving feedback from one’s peers. The use of electronic means for administration, collection and reporting of feedback was well accepted. While we did find improvement in the mean scores over time, the overall aggregate score improvements were limited due to a ceiling effect. Student feedback also suggested some uncertainty regarding the educational value of the process in enhancing professionalism.

Students and faculty do need an appreciation for the purpose and use of peer assessment to become a successful mechanism for learning. Our findings do indicate that preparing students to give and receive feedback is a key aspect in the successful delivery of peer assessment. Students are not routinely taught peer assessment skills and, like any other skill, they first need to be introduced to the concept and then allowed to practice it before they become comfortable with incorporating it into their professional behaviors. The learning advantages offered by peer assessment may be very beneficial over the course of medical education into professional practice, and longer-term study of its effect does warrant further research.

## Data Availability

The datasets analysed during the current study are available from the corresponding author on reasonable request.
